# The COVID-19 Pandemic—A Potential Role for Antivirals in Mitigating Pandemics

**DOI:** 10.3390/v15020303

**Published:** 2023-01-21

**Authors:** Gabriele Neumann, Yoshihiro Kawaoka

**Affiliations:** 1 Department of Pathobiological Sciences, University of Wisconsin-Madison, Madison, WI 53711, USA; 2 Department of Virology, Institute of Medical Science, The University of Tokyo, Tokyo 108-8639, Japan; 3 International Research Center for Infectious Diseases, Institute of Medical Science, The University of Tokyo, Tokyo 108-8639, Japan; 4 Pandemic Preparedness, Infection and Advanced Research Center, The University of Tokyo, Tokyo 108-8639, Japan

**Keywords:** SARS-CoV-2, COVID-19, pandemics, vaccines, antivirals, exit strategies

## Abstract

The COVID-19 pandemic has served as a stark reminder that outbreaks of novel pathogens (i.e., those not previously encountered by humans) have always plagued mankind and will continue to do so. The COVID-19 pandemic has also taught us that a single exposure to a novel pathogen is typically not sufficient to build robust population immunity that exists against common respiratory viruses. Robust population-level immunity can be achieved through repeated natural infection (typically at the cost of high mortality and overwhelmed public health resources) and/or repeated vaccination (which may be limited by vaccine availability, a country’s economic resources, and/or vaccine hesitancy). Here, we suggest that the broader use of antivirals could be a mitigation strategy to limit severe disease and the burden on healthcare systems during widespread virus circulation while allowing the buildup of population immunity.

## 1. Introduction

The COVID-19 pandemic started in late 2019 and the causative agent, SARS-CoV-2, spread around the world rapidly; by mid-January 2023, more than 666 million cases and more than 6.7 million deaths had occurred (https://coronavirus.jhu.edu/map.html; accessed on 13 January 2023). To date, several variants have emerged, which replaced the previously circulating strains; some of these variants are of concern because they ‘escaped’ from the immunity conferred by earlier SARS-CoV-2 infection or vaccination [1]. With most of the world transitioning from the pandemic to an endemic phase of the COVID-19 pandemic, key lessons have been learned that may help to reduce the impact of future pandemics. One such lesson is that to transition out of a pandemic, multiple exposures to the novel pathogen are likely necessary to establish robust population-level immunity similar to that against common human respiratory viruses. Several strategies can induce this kind of robust population-level immunity. Vaccination (once vaccines to a novel pandemic virus are available) is the most common, and in the long run most effective, strategy to build population-level immunity against novel viruses, but the vaccine efficacy may be lower than desired, and vaccine supplies may be limited, especially during the early phases of a pandemic. Broad population-level immunity can also be achieved through (repeated) natural infections with the novel pandemic pathogen and variants that may arise quickly. Natural infections with several variants would be ideal to elicit robust immunity, but the uncontrolled spread of highly transmissible and potentially highly pathogenic viruses in naïve populations can easily overburden healthcare systems and lead to high morbidity and mortality. Excess morbidity and mortality are typically highest in the elderly, young children, pregnant women, and individuals with underlying health issues, such as immunodeficiencies, obesity, diabetes, or cardiovascular disease. A combination of both scenarios (i.e., vaccination and natural infections) is most realistic; in fact, for COVID-19, this occurred in many countries. Another strategy, not widely employed during the COVID-19 pandemic, would be to ‘allow’ widespread natural infections while reducing the negative consequences (including excess morbidity and mortality) through the broad use of antivirals. This strategy could provide a balance between the rapid building of population-level immunity through natural infections while reducing some of the dire consequences of rapid virus spread in naïve populations.

## 2. Lessons Learned from Common Human Respiratory Viruses

By the age of 10, most children have experienced at least one infection with a common human respiratory virus, such as influenza [2], rhino- [3], respiratory syncytial [4], parainfluenza [5], human metapneumo- [4], adeno- [6], boca- [7], or human coronaviruses [8]. Due to waning immunity and/or the emergence of novel antigenic variants, reinfections frequently occur. For the four common human coronaviruses (i.e., HCoV-229E, HCoV-NL63, HCoV-OC43, and HCoV-HKU1), first infections occur during childhood and reinfections are common throughout life [9,10,11,12,13,14,15,16]. Serum antibody titers wane appreciably within one year of infection, leading to frequent reinfections with a homologous virus [9,10,17,18]. Reinfections with a homologous HCoV have even been detected in the presence of neutralizing antibody titers in serum [17]; however, such reinfections tend to cause limited clinical symptoms [9,17], suggesting that repeated infections with common human coronaviruses may lead to B- and/or T-cell responses that do not prevent reinfection, but protect against severe clinical symptoms.

At some point, many of the now common human respiratory viruses may have been pandemic viruses. Examples are the pandemic influenza viruses from 1918, 1957, 1968, and 2009. These pandemics were caused by wholly avian influenza viruses or reassortants between human and non-human influenza viruses that infected humans and spread rapidly in naïve human populations that had not been exposed to a virus with similar antigenic properties. In addition, an H1N1 influenza virus that was antigenically similar to viruses circulating in the 1950s caused an outbreak in 1977. Despite increasing immunity in human populations through natural infection and vaccination, the pandemic viruses were not eliminated from human populations but became common respiratory pathogens. Similarly, the four common human coronaviruses (HCoV-229E, HCoV-NL63, HCoV-OC43, and HCoV-HKU1) may have been introduced from an animal reservoir into humans and eventually became endemic in their new host. SARS-CoV-2 is also believed to have originated from an animal reservoir [19,20,21] and we expect a trajectory similar to that of other common respiratory viruses; that is, with increasing population-level immunity, SARS-CoV-2 will likely become a common human respiratory virus that causes repeated infections with limited morbidity and mortality, although high-risk groups (such as the elderly) may experience more severe disease than the general public. Like influenza viruses, repeated infections with antigenically distinct SARS-CoV-2 viruses may occur throughout life, and SARS-CoV-2 vaccines may need to be updated periodically to reflect the changing antigenic properties of the circulating variants.

## 3. Building Population Immunity

As outlined above, multiple exposures to SARS-CoV-2 (through vaccination and/or natural exposure) may be necessary to build population immunity similar to that against common respiratory viruses (with reinfections throughout life causing mostly asymptomatic or mild infections). This concept is supported by data showing that hybrid immunity from SARS-CoV-2 vaccination and natural infection results in more robust and broader immunity than either vaccination or infection only [22,23,24,25].

**Figure 1:**
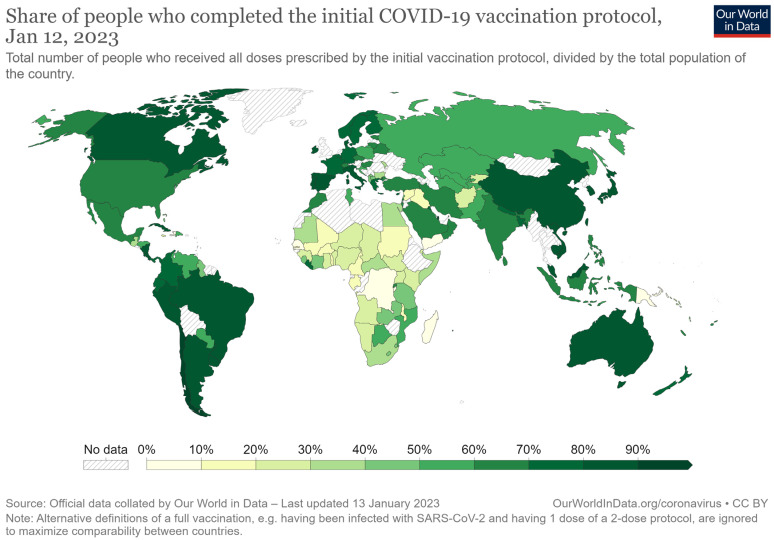
Percentages of people who have completed the vaccination protocol [28].

By the end of 2022, more than 13 billion doses of COVID-19 vaccine had been administered, yet about one-third of the global population remains unvaccinated [26]. Only few countries have reported vaccination rates of greater than 90% (including Portugal, Chile, Vietnam, Singapore, Argentina, and Canada), whereas a larger number of countries have vaccinated more than 60% of their population (Figure 1). It should be noted that even countries with ample vaccine supply and free vaccinations have not achieved the desired vaccination rate, primarily because of vaccine hesitancy (these countries include the US and several European countries) [27]. In other countries, often those with limited economic resources, the vaccination rate remains below 30% (Figure 1). However, vaccination rates are difficult to compare because the different SARS-CoV-2 vaccines require different numbers of doses.

Based on a meta-analysis of 965 seroprevalence studies, the global seroprevalence rate (reflecting both vaccination and natural infection) was 59.2% in September 2021 [29] and has since risen to greater than 80% in many countries due to the Omicron wave [29]; however, many people have not yet experienced multiple exposures to multiple SARS-CoV-2 variants. Moreover, the seroprevalence rates differ greatly among countries.

Countries with limited financial, economic, and healthcare resources did not implement comprehensive measures to contain or eliminate COVID-19. In Africa, the infection-induced seroprevalence rate rose from <5% in the spring of 2020 [30] to more than 86% by the end of 2021 [29]. Vaccines to SARS-CoV-2 only became available after most people had been naturally infected [31]. These populations may be developing immunity similar to that against common human respiratory viruses. Nevertheless, the unchecked spread of SARS-CoV-2 should not be the preferred strategy because of the negative consequences outlined earlier.

At the beginning of the pandemic, many counties (primarily those in North America, Europe, and parts of Asia) tried to manage the pandemic through a combination of non-pharmaceutical interventions and vaccination (once vaccines became available). With increasing vaccination rates and a large Omicron wave in early 2022, the overall seroprevalence rates in most of these countries are now above 90% [29,32], and most, but not all, non-pharmaceutical interventions have been lifted. By mid-November 2022, over 94% of the US population was estimated to have been infected with SARS-CoV-2 at least once, and half of the population was estimated to have been infected more than once [33]. Even so, most people may not have experienced multiple exposures with different variants to develop broad B- and T-cell immune responses.

Countries, such as Japan, Australia, New Zealand, and China, implemented stringent border-closings, a measure that in some countries was combined with extensive domestic contact tracing. (Partial) Border openings in Australia, New Zealand, and Japan in 2022 resulted in increased numbers of natural infections. In Japan, seroprevalence to infection increased from 5% in March 2022 [34] to 28.6% (95%CI: 27.6–29.6%) by the end of October 2022 (https://www.niid.go.jp/niid/images/epi/corona/82/covid19-82.pdf); similar to that in other countries, seroprevalence was higher in younger than in older people. In New Zealand, seroprevalence increased after the border was opened to roughly 31% in July 2022 [35] and 41% by the end of 2022 (https://www.health.govt.nz/covid-19-novel-coronavirus/covid-19-data-and-statistics/covid-19-case-demographics; accessed on 7 January 2023). The vaccination rates in these countries are greater than 80%, but the relatively low rates of natural infections suggest that most of their citizens will not yet have robust immunity to SARS-CoV-2.

During the first two years of the COVID-19 pandemic, China’s ‘Zero Covid Strategy’ (with strict travel restrictions, lengthy lockdowns, mandatory testing, and vaccination campaigns) prevented the extensive spread of the novel pandemic virus in China, resulting in relatively low numbers of COVID-19 infections and fatalities. However, the highly transmissible Omicron variants proved more difficult to contain and caused more frequent and larger outbreaks in different parts of the country. Consequently, when China ended its strict containment measures in December 2022, the number of COVID-19 infections in China increased rapidly. While the overall vaccination rate in China is greater than 85% [36], it is much lower among the elderly [36], leaving many people at high risk of severe COVID-19 infections. However, the currently circulating Omicron variants are less pathogenic (though more transmissible) than the ancestral SARS-CoV-2 virus, and thus, China may not encounter case fatality rates that are as high as those encountered by other countries during the early phase of the pandemic.

## 4. Exit Strategies from Pandemics

Antivirals have not received much attention in the management of pandemics. Here, we posit that antivirals could be a tool to mitigate severe disease while allowing immunity to build in humans. Remdesivir (a SARS-CoV-2 polymerase inhibitor) is approved for the treatment of COVID-19 by the US Food and Drug Administration (FDA), but its intravenous administration limits its use in outpatient settings. Molnupiravir (Lagevrio), a ribonucleoside analogue, has received Emergency Use Authorization by the FDA. In a clinical trial, molnupiravir provided a relative risk reduction of 30% from COVID-19-related hospitalization or death [37]. Paxlovid (a SARS-CoV-2 protease inhibitor composed of two compounds, nirmatrelvir and ritonavir) received EUA approval in December of 2021 for the treatment of mild-to-moderate COVID-19 in adults and children aged 12 years or older. In clinical trials, Paxlovid reduced the risk of hospitalization and death due to COVID-19 by 89% [38]. Both antivirals are administered orally, thus allowing at-home treatment. Nonetheless, these antivirals have not been prescribed widely; for example, as of 24 May 2022, only about 831,000 courses of Paxlovid had been prescribed in the US (https://www.fiercepharma.com/pharma/pfizers-paxlovid-use-soars-uptake-merck-az-covid-drugs-remains-limited; accessed on 6 January 2023).

In Japan, patients with influenza-like illness are tested with rapid antigen tests and if found positive are prescribed an antiviral to influenza—a similar strategy for high-risk COVID-19 patients could be envisioned to mitigate the burden of pandemics. This strategy could prevent high mortality while tolerating a certain level of virus spread in the population, thereby increasing population-level immunity. However, this strategy would require that antivirals are approved and widely available (including middle- and low-income countries). A potential concern with this strategy is the emergence of drug-resistant variants. In experimental settings, resistance to nirmatrelvir (an active component of Paxlovid) arises [39,40], but the mutations responsible have not been detected frequently in patient samples. Importantly, combination therapy with antivirals targeting different viral proteins or different steps in the viral life cycle might help reduce the emergence of drug-resistant variants. Thus, sustained vaccination efforts coupled with the broader prescription of antivirals to COVID-19 patients to improve population-level immunity without increasing mortality could present an exit strategy from the current pandemic for countries, such as Japan, for which the population has not encountered several natural infections with different variants. Moreover, such a strategy could help countries to manage the current wave of COVID-19 infections without overwhelming their healthcare systems and causing appreciable numbers of deaths. The use of antivirals to manage pandemics may have the added advantage that many people may be more willing to take a pill than to get vaccinated.
